# Supplementary material for: End Sequence Analysis Toolkit (ESAT) expands the extractable information from single-cell RNA-seq data
Source: Genome Res. 2016 Oct;26(10):1397–410. doi: 10.1101/gr.207902.116 (PMC5052061; doi:10.1101/gr.207902.116)
Supplement: Supplemental Material [file supp_gr.207902.116_Supp_Materials.tar.gz › ESAT-esat_v0.1/doc/html/class_jama_1_1_eigenvalue_decomposition.html]

ESAT: Jama.EigenvalueDecomposition Class Reference


ESAT


- Main Page
- Related Pages
- Packages
- Classes
- Files

- Class List
- Class Index
- Class Hierarchy
- Class Members

All Classes Namespaces Files Functions Variables Enumerator Pages

- Jama
- EigenvalueDecomposition

Public Member Functions |
List of all members

Jama.EigenvalueDecomposition Class Reference

Inheritance diagram for Jama.EigenvalueDecomposition:

[legend]

Collaboration diagram for Jama.EigenvalueDecomposition:

[legend]

|  |  |
| --- | --- |
| Public Member Functions | |
|  | EigenvalueDecomposition (Matrix Arg) |
|  | |
| Matrix | getV () |
|  | |
| double[] | getRealEigenvalues () |
|  | |
| double[] | getImagEigenvalues () |
|  | |
| Matrix | getD () |
|  | |

## Detailed Description

Eigenvalues and eigenvectors of a real matrix.

If A is symmetric, then A = V\*D\*V' where the eigenvalue matrix D is diagonal and the eigenvector matrix V is orthogonal. I.e. A = V.times(D.times(V.transpose())) and V.times(V.transpose()) equals the identity matrix.

If A is not symmetric, then the eigenvalue matrix D is block diagonal with the real eigenvalues in 1-by-1 blocks and any complex eigenvalues, lambda + i\*mu, in 2-by-2 blocks, [lambda, mu; -mu, lambda]. The columns of V represent the eigenvectors in the sense that A\*V = V\*D, i.e. A.times(V) equals V.times(D). The matrix V may be badly conditioned, or even singular, so the validity of the equation A = V\*D\*inverse(V) depends upon V.cond().

## Constructor & Destructor Documentation

|  |  |  |  |  |  |
| --- | --- | --- | --- | --- | --- |
| Jama.EigenvalueDecomposition.EigenvalueDecomposition | ( | Matrix | *Arg* | ) |  |

Check for symmetry, then construct the eigenvalue decomposition

Parameters
:   |  |  |
    | --- | --- |
    | A | Square matrix |

Returns
:   Structure to access D and V.

## Member Function Documentation

|  |  |  |  |  |
| --- | --- | --- | --- | --- |
| Matrix Jama.EigenvalueDecomposition.getD | ( |  | ) |  |

Return the block diagonal eigenvalue matrix

Returns
:   D

|  |  |  |  |  |
| --- | --- | --- | --- | --- |
| double [] Jama.EigenvalueDecomposition.getImagEigenvalues | ( |  | ) |  |

Return the imaginary parts of the eigenvalues

Returns
:   imag(diag(D))

|  |  |  |  |  |
| --- | --- | --- | --- | --- |
| double [] Jama.EigenvalueDecomposition.getRealEigenvalues | ( |  | ) |  |

Return the real parts of the eigenvalues

Returns
:   real(diag(D))

|  |  |  |  |  |
| --- | --- | --- | --- | --- |
| Matrix Jama.EigenvalueDecomposition.getV | ( |  | ) |  |

Return the eigenvector matrix

Returns
:   V

---

The documentation for this class was generated from the following file:

- java/Jama/EigenvalueDecomposition.java


---

Generated on Fri Aug 15 2014 15:11:06 for ESAT by  

 1.8.7
